# Estimating food portions. Influence of unit number, meal type and energy density^☆☆^^☆☆^

**DOI:** 10.1016/j.appet.2013.07.012

**Published:** 2013-12-01

**Authors:** Eva Almiron-Roig, Ivonne Solis-Trapala, Jessica Dodd, Susan A. Jebb

**Affiliations:** a MRC Human Nutrition Research, Elsie Widdowson Laboratory, Fulbourn Road, Cambridge CB1 9NL, UK; b Department of Clinical Sciences, University of Chester, Parkgate Road, Chester CH1 4BJ, UK

**Keywords:** Portion size estimation, Unit number, Snack, Meal, Energy density

## Abstract

Estimating how much is appropriate to consume can be difficult, especially for foods presented in multiple units, those with ambiguous energy content and for snacks. This study tested the hypothesis that the number of units (single vs. multi-unit), meal type and food energy density disrupts accurate estimates of portion size. Thirty-two healthy weight men and women attended the laboratory on 3 separate occasions to assess the number of portions contained in 33 foods or beverages of varying energy density (1.7–26.8 kJ/g). Items included 12 multi-unit and 21 single unit foods; 13 were labelled “meal”, 4 “drink” and 16 “snack”. Departures in portion estimates from reference amounts were analysed with negative binomial regression. Overall participants tended to underestimate the number of portions displayed. Males showed greater errors in estimation than females (*p* = 0.01). Single unit foods and those labelled as ‘meal’ or ‘beverage’ were estimated with greater error than multi-unit and ‘snack’ foods (*p* = 0.02 and *p* < 0.001 respectively). The number of portions of high energy density foods was overestimated while the number of portions of beverages and medium energy density foods were underestimated by 30–46%. In conclusion, participants tended to underestimate the reference portion size for a range of food and beverages, especially single unit foods and foods of low energy density and, unexpectedly, overestimated the reference portion of high energy density items. There is a need for better consumer education of appropriate portion sizes to aid adherence to a healthy diet.

## Highlights

• University staff/students showed poor awareness of appropriate portion sizes.
• Unit number and meal classification affected portion size estimation.
• Portion sizes in sugary drinks, pizza and pasta were underestimated by 30–46%.
• Women were better at estimating food portion sizes than men.

## Background

Large portions, in particular of highly palatable foods, challenge innate human appetite control systems and may lead to weight gain (Prentice & Jebb, 2003; Wansink & Van Ittersum, 2007). This phenonemon has been reported in controlled studies across a wide range of foods and participant characteristics (Jeffery et al., 2007; Ledickwe, Ello-Martin, & Rolls, 2005; Rolls, Roe, Kral, Meengs, & Wall, 2004; Rolls, Roe, & Meengs, 2007; Rolls, Roe, Meengs, & Wall, 2004), as well as in population studies (Duffey & Popkin, 2011; Kelly et al., 2009).

Estimating how much is appropriate to consume can be difficult. Research suggests that appropriate portion sizes of some items can be particularly hard to judge, such as highly palatable foods with low satiating effects (Prentice & Jebb, 2003; Yeomans, Blundell, & Leshem, 2004), energy-dense foods (Anderson et al., 2008; Carels, Konrad, & Harper, 2007; Japur & Diez-Garcia, 2010) amorphous foods (i.e. those who take the shape of the container they are in) (Slawson & Eck, 1997), foods presented in large packs or containers (Marchiori, Corneille, & Klein, 2012; Raynor & Wing, 2007; Wansink & Kim, 2005) and foods made up of multiple units like certain snacks, restaurant meals and on-the-go “meal deals” (Kral, 2006; Wansink, 1996).

Previous studies into portion size estimation have focused on single unit foods which tend to be overconsumed, something known as “unit bias” (Brogden & Almiron-Roig, 2011; Hernandez et al., 2006; Japur & Diez-Garcia, 2010; Rolls, 2003). According to this theory, the mere presentation of a food as a single entity can lead it to be considered as the appropriate amount to consume independently of other food attributes (Geier, Rozin, & Doros, 2006), and lead to overconsumption if the size of the unit is large (Young & Nestle, 2002). The ‘appropriate amount’ may be determined by what is culturally accepted as a standard unit, such as what is consumed regularly at home or in a restaurant, or bought from the store, and thus subject to social norms (Wansink & Van Ittersum, 2007). Laboratory studies have confirmed that unit bias occurs and leads to increased consumption. When study participants were asked to compare the portion size of various sandwiches (presented as a single unit) to the usual portion of the same food, they rated the portions of the larger sandwiches as larger than their usual portion of sandwich (Rolls, Roe, Meengs, et al., 2004). However this cognitive perception of a volume difference relative to a usual amount was not sufficient to control intake of the larger sandwiches, and the more that was presented the more food was consumed.

On the other hand, when the food is composed of multiple units, whether of the same repeated units or of varied items (referred as “multi-unit” food in both cases), its total portion size could also be difficult to estimate, especially if the various items are different in nature (Wansink & Van Ittersum, 2007). It is thought that when absolute size of food increases, sensitivity to size is reduced (Chandon & Ordabayeva, 2009; Chandon & Wansink, 2002), especially when changes occur in the three dimensions (Krishna, 2007) as happens with increasing unit number. The presence of multiple items in a meal may lead consumers to rely on total volume or other dimensional aspects of the meal rather than food type or energy content (Geier & Rozin, 2009). Studies have shown that increasing the portion size of multi-unit meals including a main course with starch and vegetable side dishes results in increased cumulative intake (Rolls et al., 2007). Similar results were obtained for multi-unit savoury snacks (Raynor & Wing, 2007) and intended consumption of sweets (Wansink, 1996). Overall it seems that both unit bias and multi-unit presentation could lead to overconsumption.

Beyond unit number, the food’s energy content may also influence judgements about portion size. It has been postulated that incorrect portion estimation for high fat/high energy foods may be associated with a progressive inability to correctly evaluate the energy and fat content with increasing portion size (Rolls et al., 2007; Wansink & Van Ittersum, 2007). Indeed, some high energy density foods such as sweet and savory snacks, pizza, fried potatoes and desserts are often chosen in significantly larger portions than recommended perhaps because they are expected to be less satiating (Brunstrom, Shakeshaft, & Scott-Samuel, 2008; Burger, Kern, & Coleman, 2007) and their portion sizes are poorly estimated (Anderson et al., 2008; Japur & Diez-Garcia, 2010; Yuhas, Bolland, & Bolland, 1989). In addition, some of these foods may be considered ‘unhealthy’ by some individuals which may lead them to overestimate appropriate portion sizes based on the perceived amount of energy (Carels et al., 2007; Kelly et al., 2008). In a previous study (Brogden & Almiron-Roig, 2011) energy density explained 13% of the variance in percent error of estimation and positively correlated with a high error in estimation vs. reference amounts across a sample of 8 foods. This association was dependent on the energy content more than the volume of the foods presented. However the results may have been affected by the presence of beverages (high energy content and low energy density) and the small number of items explored.

Finally, evidence suggests that the perception of an item as being a beverage, a meal or a snack may trigger certain cognitive processes that affect how much we chose to consume (Capaldi, Owens, & Privitera, 2006; Shimizu, Payne, & Wansink, 2010). For instance, when presented with a food, participants eat more if they perceive the food to be a meal as opposed to a snack, but only when they are hungry (Shimizu et al., 2010). This suggests that some cognitive effects are strong enough to alter the physiological satiety response. This was confirmed in another study where differences in the expected physical form of the food once in the stomach were associated with changes in gastric emptying and orocecal transit times (Cassady, Considine, & Mattes, 2012). Whether such mechanisms also occur through portion size perception effects is not known.

This study explored the effect of three specific food attributes, unit number, meal type and energy density (ED) on estimated portion size (as portion number), using a sample of popular foods and beverages available from a university campus. We asked participants to quantify the number of portions based on habitual consumption, of 33 different foods and drinks displayed to them. We then compared their answers against the corresponding number of portions based on a reference instrument from the U.K. Food Standards Agency (based on customary consumption and used for food labelling) (FSA, 2002). Our main study hypothesis was that the number of portions of single unit foods and that of meals and beverages would be estimated closer to the reference number of portions than the portion size of multi-unit foods and of snacks. We also hypothesized that the number of portion sizes for high energy density foods would be estimated as fewer than the corresponding reference number of portions and that estimations would be influenced by the perceived fat and energy content (Rolls et al., 2007). Methods based on habitual consumption contain a subjective component and may be influenced by learning or experience with that food, which may make them more user-friendly but less sensitive for estimating portions of foods composed of various units, than other methods (Kral, 2006). We therefore explored participants’ ability to estimate portions using two previously piloted methods, rating the number of portions displayed (Brogden & Almiron-Roig, 2011) and a scale-based rating of the portion amount against habitual amounts (Kral, 2006). Evidence suggests that men and women estimate portion sizes differently (Brunstrom et al., 2008; Burger et al., 2007), therefore our analyses were adjusted for gender differences.

## Methods

### Participants

A sample of 32 individuals was recruited from the University of Chester campus and surrounding area for a study “exploring portion sizes of commonly consumed foods”. Sample size was based on previous studies (Blake, Guthrie, & Smicklas-Wright, 1989; Brogden & Almiron-Roig, 2011), and included a repeated measures design to increase sensitivity. Eligibility criteria included: 18–45 years; a BMI between 18.5 and 27 kg/m^2^; non-dieting; non-smoking and consuming breakfast regularly. Only non-obese subjects were included to decrease variability in portion size estimation (Kelly et al., 2008). Exclusion criteria included: conditions affecting diet or appetite; food allergies or intolerances to the study foods; prolonged weight cycling; medications/supplements affecting appetite; performing >10 h per week of intense physical activity; disliking or being unfamiliar with >50% of the food/drink items; and a relevant qualification in nutrition. Individuals wishing to participate in the study were pre-screened via a telephone interview after which their weight and height were confirmed in the laboratory. Candidates completed the Three Factor Eating Questionnaire (TFEQ) (Stunkard & Messick, 1985) plus a liking and familiarity questionnaire. Based on previous work (Brogden & Almiron-Roig, 2010; Stunkard & Messick, 1985) those who scored ⩾9 on the disinhibition scale of the TFEQ or those who scored ⩾10 on the cognitive restraint scale plus ⩾7 on the hunger scale were excluded, as these individuals tend to respond to food cues differently from the general population (Blundell et al., 2008; Yeomans, Tovey, Tinley, & Haynes, 2004). Liking and familiarity were assessed on 100 mm visual analogue scales. Participants were excluded if they scored <50 mm (liking) or <50 mm (familiarity) on 17 or more of the study foods (Raudenbush & Frank, 1999). After applying the exclusion criteria, a total of 15 men and 17 women (all British white) were enrolled and completed the study. Mean BMI (±SD) was 23.9 ± 2.7 kg/m^2^ for the whole group, 25.1 ± 2.5 for males and 22.8 ± 2.5 for females. Mean age (±SD) was 25.0 ± 5.9 years for the whole group, 27.4 ± 7.6 for males and 22.7 ± 2.5 for females. Mean dietary restraint, disinhibition and hunger scores (±SD) were 4.5 ± 2.7, 4.1 ± 1.6 and 5.3 ± 2.0, respectively for the whole group, 3.4 ± 2.9, 4.1 ± 2.1 and 6.0 ± 2.2 for males; and 5.4 ± 2.2, 4.1 ± 1.2 and 4.8 ± 1.8 for females.

### Study design

This was a within-subject, repeated measures study with each participant returning for three separate test sessions, spaced at least 7 days apart. Each subject rated the number of portions, fat content and energy content for 11 different foods and/or drinks at each visit (total *n* = 33 foods), using a questionnaire. Foods were allocated to one of 3 sessions using a random sequence with 11 foods per session. Each food was assigned a reference number of portions based on the Food Standards Agency portion size scheme (FSA, 2002). Thus the number of reference portions ranged between 0.5 and 5.3, with energy density values between 1.7–26.8 kJ/g. Foods included a variety of single unit and multi-unit foods, which may be typically consumed as meals, beverages or snacks and labelled accordingly. In order to standardise baseline appetite levels participants were asked to consume their usual breakfast at home and a mid-morning snack provided, 2 h before the test, on each study day. The study protocol was approved by the Faculty of Applied and Health Sciences ethics committee, University of Chester. All participants provided consent and received a £20 supermarket voucher for their participation.

### Procedures

Participants reported to the laboratory at 12.00 noon after consumption of their usual breakfast at home at 08:30 am, followed by a snack (Nestlé, KitKat Chunky, 48 g) at 10:30 am, after which they fasted until arrival to the laboratory (except for water). Participants were asked to refrain from drinking alcohol and to keep evening meals and activity levels similar on the day before each test. To enhance compliance, food intake and physical activity was monitored on arrival using a conditions check questionnaire (Brogden & Almiron-Roig, 2011). Subsequently participants completed baseline appetite ratings using visual analogue scales (VAS) (see ‘Appetite ratings’), and were allowed a few minutes to read the study questionnaire. Participants were then verbally reminded of the definition of a portion as “the quantity of food/drink that you would consume on one eating/drinking occasion” i.e. at that moment in time and to consider this definition when completing the questionnaire (Schwartz & Byrd-Bredbenner, 2006). Four questions (see ‘Portion, energy and fat estimates’) were presented as a booklet with one question per page. Participants were asked to respond to each question without considering their answer to the previous question and to avoid talking amongst themselves during the test. At 12:25 pm participants moved to the test room, located their starting booth and started the test. Subjects were given a minute at each booth to complete the questionnaire, they then rotated clockwise and repeated the process for all 11 booths. Before leaving, each participant received the free snack for the following session. On their final session, participants received the gift voucher and were offered the opportunity to ask questions regarding the study.

### Appetite ratings

Participants rated baseline hunger, fullness and thirst levels using validated 100 mm visual analogue scales (VAS) (Flint, Raben, Blundell, & Astrup, 2000), presented in booklet form, one scale per page and anchored at each end with opposite labels. Thus, for the question ‘How (attribute, e.g. hungry) do you feel?’ the scale ranged from ‘not (attribute) at all’ to ‘extremely (attribute)’. These ratings were used to confirm standardised appetite levels before each test (Brogden & Almiron-Roig, 2011). In addition to the appetite questions, three distraction questions (on alertness, tiredness and sleepiness) were inserted to diminish experimental bias. The data from the distracting questions were not analysed.

### Portion, energy and fat estimates

Participants completed one full questionnaire for each food, consisting of four questions, which were presented in randomised order across subjects. Participants rated the number of portions of food displayed in front of them by answering to the question “how many portions of (food/drink) are in this (container type)” (Brogden & Almiron-Roig, 2011). Container type included plate, bowl, tub, cup or pack. Portions could be recorded as a full number or as a fraction, for example 0.5, 1 or 1.5 portions. Participants rated fat and energy content on 100 mm VAS preceded by “how much fat do you think this portion of X contains?” anchored with “no fat at all” to “extremely high in fat”; and “how many calories do you think this portion of X contains?”, anchored with “no calories at all” to “extremely high in calories”, as previously reported (Rolls et al., 2007). Participants also rated how the displayed portion compared to their habitual portion using a 100 mm VAS preceded by the question “how does this serving compare to your usual portion of X food/drink?” (“a lot smaller” to “a lot larger”) (Kral, 2006).

### Test foods and beverages

The test foods included meals, snacks and beverages typically consumed by the university population (Table 1). Foods consisting of a single item (such as one sausage roll) or presented as one homogenous mixture (such as a meal of macaroni and cheese) were classified as ‘single unit’ foods (*n* = 21). Foods consisting of more than one repeated item (such as cheese and crackers) or a combination of different items (such as meat pie with two side vegetables) were classified as ‘multi-unit’ foods (*n* = 12). The smaller number of multi-unit foods was due to the difficulty in identifying multi-unit foods relevant for our study population that included a broad range of energy densities and that could be eaten across a range of meal contexts. Single unit items included four energy-yielding beverages, labelled “drink”, and presented in their original containers as sold. Thirteen meals were presented on a dinner dish (25 cm diameter) and labelled “meal”, and 16 snacks were presented on a dessert dish (16 cm diameter), bowls (14.5–20 cm diameter, 4–9 cm deep) or original container and labelled “snack”. For the breakfast cereal with milk, the cereal was presented in a breakfast bowl and the milk in a tall glass (6 cm diameter, 13 cm depth). To decrease variability between subjects in their interpretation of the food item as meals or snacks (Wadhera & Capaldi, 2012) each item was labelled to indicate whether it should be considered a meal, a beverage or a snack depending on the suitability of that food at the time of the test, which was always at lunch time. Five of the 33 foods were of very low energy density (ED) (0–2.5 kJ/g), 10 foods were of low ED (2.5–6.3 kJ/g), 9 foods were of medium ED (6.3–16.7 kJ/g), and 9 foods were of high ED (>16.7 kJ/g) (Rolls & Barnett, 2000). Amounts displayed for each item were based on manufacturer’s guidelines for one portion or container size, usually provided on the pack. The pizza portion corresponded to a standard pre-cut portion from a popular retailer (160 g). Each item was presented in an individual booth, with the sequence of presentation randomized across sessions. All visible brand names, weight and/or nutritional information were disguised. Foods requiring cooking or heating were prepared as instructed by the manufacturer, then cooled and displayed with a plate cover to decrease odour release.

### Comparison with reference portions

The estimated number of portions given by participants was compared with the reference number of portions based on the Food Standards Agency reference instrument (FSA, 2002). This instrument is based on amounts customarily consumed per eating occasion by the general population and was previously shown to be a more readily interpreted instrument than other tools in this study population (i.e. healthy subjects) (Brogden & Almiron-Roig, 2011). Each displayed amount of food was assigned a reference number of portions (FSA, 2002) (Table 1). For multi-unit foods not listed in the FSA portion size reference book the reference number of portions was calculated from the sum of the portions for each food item in the meal (by FSA standards) divided by the number of items in the meal. For example, for the cheese and crackers, for which we displayed a portion size of 30 and 25 g of cheese and crackers respectively, the cheese corresponded to 0.75 of the reference amount (40 g) and the crackers corresponded to 0.76 of the reference amount (33 g), so the total reference portion for this food was (0.75 + 0.76)/2, or 0.75.

### Statistical analysis

The primary aim of the analyses was to investigate the effects of food attributes on the participants’ accuracy at estimating the number of portions compared with FSA reference values. Estimates of the number of portions for 33 foods were collected from 32 subjects, generating 1056 observations. To quantify the degree of departure of each portion estimate vs. its corresponding reference portion we used the ratio of the estimated portion and its reference value. This ratio hereafter referred to as PER (*portion estimation ratio*) provides a measure of the departure in the number of estimated vs. reference portions, with values >1 indicating over-estimation, values close to 1 indicating accurate estimation, and values <1 representing under-estimation.

The PER can be converted into % error of estimation (Blake et al., 1989; Brogden & Almiron-Roig, 2011) by applying the following conversion: % Error = 100 * (PER−1).

Simple methods of analysis for the PER data, for example standard linear regression, are not applicable to this study because the observations are not independent due to the repeated-measures design, and because the assumption of normality in portion estimates is often problematic. To circumvent these problems we multiplied the estimated number of portions by 100 to yield an integer number greater than 0 which can be fitted using regression models suitable for counts and thus the normality assumption is not necessary.

To take into account that measurements within the same individual may be correlated, we fitted a multivariate negative binomial regression model suitable for repeated count data to the transformed number of estimated portions (Solis-Trapala & Farewell, 2005). This model is characterised by mean functions and a parameter that models the correlation between repeated observations per subject (33 foods) and *overdispersion* (when the observed variance is higher that the variance of the theoretical model), which is common in count data. We express the Log(100 × Mean number of Estimated Portions) in terms of explanatory variables (*x_ij_*) and their interactions. The reference amount is also specified in the linear predictor as an *offset* for Log (100 × number of Reference Portions) with a regression coefficient constrained to 1, as follows:

$$\text{Log}(100\times {\text{Mean number of Estimated Portions}}_{\mathrm{ij}})=\text{Log}(100\times \text{number of Reference Portions})+x_{\mathrm{ij}}^{\prime }B$$

which is equivalent to

$$\text{Log}(100\times {\text{Mean number of Estimated Portions}}_{\mathrm{ij}})-\text{Log}(100\times \text{number of Reference Portion})=\text{Log}(\text{Mean}({\text{PER}}_{\mathrm{ij}}))=x_{\mathrm{ij}}^{\prime }B$$

,where *i* indexes participant, *i* = 1, …, 32; *j* indexes foods, *j* = 1, …, 33 and *B* are regression coefficients. These coefficients are the parameters of interest and quantify the difference of the log mean estimated PERs for a unit change in the explanatory variable. The method used to estimate the parameters is robust to distributional assumptions (Solis-Trapala & Farewell, 2005).

Separate regression models were first built to investigate the effects of unit number category (multi-unit vs. single unit), meal type assignation (snacks vs. other, hereafter referred as ‘label’), energy density, estimated energy, and estimated fat content on mean PER. Given the possible difference in portion size estimation between genders (Burger et al., 2007) this was also investigated in a separate model. We then developed a joint model including interactions and assessed the effect of order of food exposure plus checked model adequacy through plots of residuals and sensitivity analysis to outliers.

Mean portion estimates by food were compared against reference amounts with *t*-tests. VAS ratings for habitual portion size comparisons were summarised with means and 95% CI, where a 50 mm value was considered to signify ‘same size as usual portion’.

To confirm adherence to the protocol, mean baseline appetite ratings across study sessions were compared using one-way repeated measures ANOVA.

## Results

### Effect of unit number, label, energy density, and perceived energy and fat content on number of estimated portions

Analyses based on the multivariate negative binomial regression confirmed that overall participants tended to underestimate the number of portions vs. the reference number of portions, with males showing greater error of estimation than females (*p* = 0.011) (Table 2, *Model 1*). Results also showed that the average portion estimation ratio (PER) was closer to 1 (that is, the number of estimated portions were closer to the reference amount) in multi-unit than in single unit foods (*p* = 0.02), and in snacks than in meals or beverages (*p* < 0.001) (*Models 2 and 3*).

In regards to energy density, the PER increased for foods with higher ED, with a 2.4% increase in PER per kJ/g (10% increase per kcal/g) (*Model 4*).

Higher levels of perceived energy (*p* = 0.021) and fat content (*p* = 0.07) led to minimal increases of PER only which although significant were not clinically meaningful (<0.3 units).

**Fig. 1:**
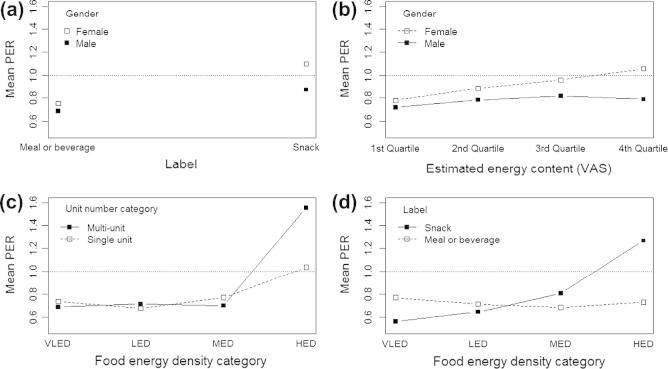
Interaction plots of mean portion estimation ratio (PER) estimated by multivariate negative binomial regression (cf. Table 2). PER = 1 indicates correct estimation, PER < 1 under-estimation and PER > 1 over-estimation, in relation to number of reference portions.

A closer exploration of the PER taking into account the various individual and food characteristics revealed important interactions (Table 2, *Model 5*). These included interactions of label with gender, energy content with gender, ED with unit number, and ED with label (Fig. 1). Both men and women underestimated the number of portions presented for meals and beverages, but men tended to underestimate also for snacks while women did not (Fig. 1a). Men underestimated portion numbers of foods to a similar extent irrespective of how much energy they perceived the foods to contain, while in women estimates of portion number improved up to the third quartile of estimated energy content, and were slightly overestimated for the top quartile (Fig. 1b). The number of portions for foods with very low, low or medium ED were equally underestimated irrespective of unit number (Fig. 1c), but estimation ratios increased for high ED foods, especially when presented as multi-unit foods. Finally, the number of portions for high ED snacks was more frequently overestimated than for meals and beverages (Fig. 1d). Label did not influence estimation of the number of portions of very low, low and medium ED foods (PER < 1). Within the high ED food category, two multi-unit foods (cheese and crackers and pork pies) and some snacks (cheese and crackers; pork pies; croissant; flapjack; peanuts) were the foods where the number of portions was most commonly overestimated (PER of ∼1.2–1.5), implying participants believed the appropriate portion size to be smaller than the reference amount.

### Number of estimated portions by participants and usual portion ratings

**Fig. 2:**
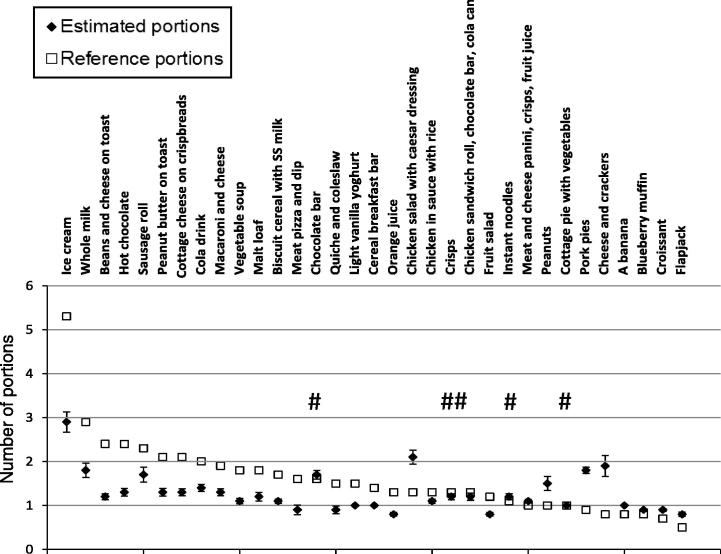
Comparison of the mean number of estimated portions (*n* = 32) with the number of reference portions across 33 food items. The *Y*-axis indicates the mean (±SEM) number of estimated portions in response to the question “how many portions of (food) are in this (container type)?”, and corresponding reference portion number. Reference portions are based on the Food Standards Agency scheme [41]. Significant differences were detected for all foods except those marked with #.

Figure 2 shows the mean number of estimated portions displayed for each food against the reference number of portions. On average, participants rated the number of portions displayed as less than the reference number of portions in 19 out of 24 foods that were presented in larger portions (>1 reference amount), while they rated the number of portions as more than (8 foods) or equal to (1 food) the reference amount in all the 9 foods that were presented as less or equal to 1 reference portion. Departures in portion estimates from reference amounts were statistically significantly different from zero at the 5% level in 28 out of the 33 foods. The instant chicken flavoured noodles, chocolate bar, potato crisps, cottage pie with vegetables and the chicken sandwich roll, chocolate bar and cola meal were the only items accurately estimated against reference amount (*p* > 0.05). Women tended to estimate the number of portions more accurately than men (data not shown). These gender differences were confirmed in the regression models (see above).

We observed that estimating the number of portions for two foods (ice cream and cheese and crackers) appeared particularly difficult for most participants, possibly due to the large container of the ice-cream and the large number of units of the cheese and crackers (8 units). Similarly we identified a participant with unusual observations (outlier). Exclusion of the ice-cream, cheese and crackers and the identified outlier did not change the results. We also adjusted for the order of food exposure, this was not statistically significant.

**Fig. 3:**
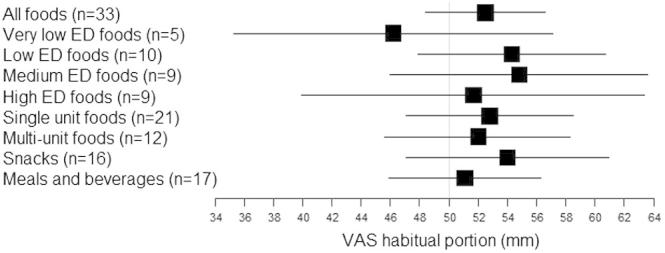
Mean and 95% confidence interval for 100 mm VAS ratings in response to the question “how does this serving compare to your usual portion of (food/drink)?”. A score of 50 mm indicates that participants found the displayed portion for a food/drink comparable to their usual portion of the same food/drink.

Participants found most of the portions displayed very close to a usual portion for that food irrespective of type of meal or unit size (95%CI crossing 50 mm in all food groups, Fig. 3).

### Baseline appetite levels

There were no significant differences in baseline hunger, fullness nor thirst within subjects across the three test days, [*F*_(2,60)_ = 0.410 for overall effect of hunger; *F*_(2,60)_ = 0.256 for fullness; *F*_(1.6,48.4)_ = 0.035 for thirst; all *p* > 0.05], or between males and females (*p* > 0.05).

## Discussion

The lean men and women in this study tended to underestimate the number of portions presented for popular meals, beverages and snacks by about 10% on average compared with a reference scheme, but there was a large variability in the portion estimates (PER 0.13-10, corresponding to −88 to 900% error) due to individual differences. Females estimated the number of portions for foods, especially snacks, with slightly less error than males and estimates in females were affected by perceived energy content, although to a very small degree (changes from 0.8 to 1.0 portion estimates). The effects of gender on portion estimation have been controversial, with some studies reporting that women are better estimators (Burger et al., 2007) and others reporting none/minimal differences (Faggiano et al., 1992; Yuhas et al., 1989). Differences across genders may reflect a function of the reference system used, or a biological response to the higher energy needs of men vs. women.

This large individual variability in portion estimation is not uncommon (Brogden & Almiron-Roig, 2011; Hernandez et al., 2006). Despite the wide range of answers, the number of estimated portions fluctuated closely above or below 1 for many foods, consistent with the concept of the unit bias effect (Geier et al., 2006). In our case, unit bias may have been facilitated by foods being presented in delimited containers or packages (Marchiori et al., 2012; Wansink & Cheney, 2005; Yuhas et al., 1989) or by the large volume and amorphous shape of some foods (Hernandez et al., 2006).

Contrary to our expectations, the number of portions for multi-unit foods was better estimated than for single unit foods. A number of these dishes were meals likely to be consumed by our study population as a single lunch item, plus were of modest size as they were displayed based on the manufacturer’s portion guidelines. Portion numbers for snacks were also better estimated than portion numbers of meals and beverages, perhaps due to people being more accustomed to consuming these foods in isolation, although in some cases (e.g. crisps, chocolate bar) differences were small.

Interestingly, label interacted with gender and ED, indicating that the perception of a food as a snack or a meal, in combination with other food attributes and subject characteristics may have implications on how well its portion is estimated. Wadhera and Capaldi (2012) reported that cheese on toast, muffins and corn crisps were equally classified as snacks or as meals by university students, while soups, burritos and pizza were nearly always considered meals. On the other hand, crackers, potato crisps and nuts were nearly always classified as snacks. It is possible that the pre-defined snack/meal classification in our study may have had an impact on the estimated number of portions (Brogden & Almiron-Roig, 2010; Brunstrom et al., 2008). For instance, foods categorized as snacks appear to be less satiating than those considered meals (Capaldi et al., 2006) and so people may consider and acceptable portion of these foods as a larger amount than what is recommended.

Discrepancies between estimated and reference number of portions were mostly independent from energy and fat content but might have been influenced by other factors not explored in the present study, such as previous exposure to large portions of the test foods (Brogden & Almiron-Roig, 2010; Brunstrom et al., 2008), habitual consumption norms (Wansink & Van Ittersum, 2007) or how healthy the food is perceived to be (Carels, Harper, & Konrad, 2006; Carels et al., 2007).

The portion estimation ratio in this study increased with ED (2.4% increase per each kJ/g or 10% increase per kcal/g), although this depended strongly on the food attributes (unit number, meal type). While the number of reference portions of very low, low and medium ED foods were mostly underestimated (PER < 0), the number of portions for high ED foods were mostly overestimated (PER > 1). Surprisingly, this implies that for the high ED foods, they perceived the appropriate portion size to be smaller than the reference portion.

Poor awareness of portion size is often cited as an explanation for overconsumption of energy dense foods (Duffey & Popkin, 2011) yet we observed the opposite phenomenon, with participants reporting that the amounts displayed were 20–50% larger than their typical portion. It is possible that our participants were aware that such foods are very energy dense (their estimated energy and fat ratings were very close to the actual content) and reported consuming a smaller portion than what they actually consume due to social desirability, awareness of their participation in a nutrition-related study or other effects. Alternatively this group of lean, university students and staff might be consuming smaller portions of such foods than the general population as part of a healthy lifestyle.

On average participants found the portion size displayed comparable to a usual portion of the same food for all foods irrespective of unit number and meal type. However they rated the number of portions for most foods as larger or smaller to what they would normally eat at one sitting (definition of a portion), and did so differently depending on unit number and meal type. This may be related to the usual portion question prompting participants to focus on the most salient features of the food such as total volume, rather than food composition and meal context.

The dissociation between recommended amounts and portions actually available to consumers has been well documented (Abramovitch et al., 2012; Rolls, 2003). Incorrect portion estimation may be associated with judgments affected by single food attributes that dominate over other dimensions (Geier & Rozin, 2009; Hernandez et al., 2006). These may include the food’s palatability (Yeomans, Blundell, et al., 2004), its expected satiating power (Brogden & Almiron-Roig, 2010), the perceived volume (Brunstrom, Collingwood, & Rogers, 2010; Hernandez et al., 2006; Raynor & Wing, 2007), and the perceived energy and fat content (Rolls et al., 2007). Although in this study some discrepancies may appear small, they may still have important effects on daily energy intakes. For instance, estimating the number of portions for energy-yielding drinks such as orange juice, whole milk, cola and hot chocolate (all very low to low ED) as 30–46% smaller than recommendations (Fig. 2) could potentially result in an extra 130–925 kJ (31–221 kcal) consumed from these drinks. On the other hand, underestimating the number of portions of the pasta dish by 32% and the pizza meal by 44% could potentially translate into an extra 515 and 1506 kJ (123 and 360 kcal) for these foods respectively, which could have important effects on weight control. Cumulatively, these errors in portion size estimation may contribute to the difficulties experienced by consumers in consuming a healthy diet.

Our study has a number of limitations. We only explored lean university staff and students of white ethnic origin which makes our results not generalizable to the broader population (Davis, Curtis, Tweed, & Patte, 2007). Participants may have estimated the number of portions on the basis of experience or familiarity (Brogden & Almiron-Roig, 2010; Brunstrom et al., 2008), which were only partially accounted in our study design (i.e. participants had to be familiar only with half of the foods to be eligible). In an attempt to mimic real-life conditions we included some pre-packaged highly palatable foods which may have challenged the participants’ ability to estimate portions (Kral, 2006; Marchiori et al., 2012; Yeomans, Blundell, et al., 2004), and which varied in volume, a factor that is known to influence expectations about foods (Brunstrom et al., 2010). Further research is needed to determine the specific effects of energy density on a larger sample of multi-unit foods, including those made of a set of varied foods as well as from repeated foods. It is also important to clarify whether estimating the number of portions above or below recommendations translates into actual different energy intakes, plus how this extends to people with higher BMI and different dietary restraint levels (Brunstrom & Mitchell, 2007).

### Conclusions

This study illustrates poor consumer awareness of appropriate portion sizes which tends towards an overestimation of the appropriate amount of food to consume. It shows that perception is influenced by the number of units, the context in which the meal is presented (meal, beverage, snack) and by characteristics of the consumer such as gender. The relationship with energy density is complex and needs further research.

## Figures and Tables

**Table 1:** Characteristics of the test foods including amount displayed, equivalent FSA portion size, energy density (ED), meal type and unit size category. ED category based on Rolls and Barnett (2000): very low, VL 0–2.5 kJ/g; low, L 2.5–6.3 kJ/g; medium, M 6.3–16.7 kJ/g; high, H > 16.7 kJ/g. Under “number of portions based on FSA reference amount”, values < 1 indicate that amount displayed (g, ml) was smaller than one reference portion; values of 1 indicate comparable portions; and values >1 indicate that the amount displayed was larger than one reference portion.

Food/drink	Amount displayed (g, ml)	Energy of portion displayed (kJ)	Number of portions based on FSA reference amount	ED category	Meal type label	Unit size presentation
Orange juice carton^a^	200 ml	339	1.3	VL	Beverage	Single unit
Pork pies (pack of 2)^b^	130 g	2232	0.9	H	Snack	Multi-unit
Cheese and crackers^c^	55 g	1028	0.8	H	Snack	Multi-unit
Cola drink^d^	500 ml	911	2.0	VL	Beverage	Single unit
Whole milk bottle^e^	568 ml	1568	2.9	L	Beverage	Single unit
Hot chocolate^f^	473 ml	2019	2.4	L	Beverage	Single unit
Peanut butter on toast (2 slices)^g^	92 g	1195	2.1	M	Meal	Multi-unit
Country vegetable soup^h^	400 g	635	1.8	VL	Meal	Single unit
Fruit salad^i^	134 g	376	1.2	L	Snack	Single unit
Light vanilla yoghurt^j^	190 g	397	1.5	VL	Snack	Single unit
A banana^k^	140 g	523	0.8	L	Snack	Single unit
Croissant^l^	44 g	786	0.7	H	Snack	Single unit
Bowl of chicken salad with caesar dressing^m^	325 g	1442	1.3	L	Meal	Single unit
Biscuit cereal with glass of milk^n^	193 g	844	1.7	L	Meal	Multi-unit
Instant noodles^o^	300 g	2195	1.1	M	Meal	Single unit
Flapjack^p^	28 g	543	0.5	H	Snack	Single unit
Chocolate bar^q^	75 g	1705	1.6	H	Snack	Single unit
Blueberry muffin^r^	70 g	1137	0.8	M	Snack	Single unit
Cereal breakfast bar^s^	45 g	1016	1.4	H	Snack	Single unit
Cottage cheese on crispbreads^t^	155 g	915	2.1	L	Snack	Multi-unit
Sausage roll^u^	140 g	2082	2.3	M	Snack	Single unit
Beans and cheese on toast (2 slices) v	522 g	2621	2.4	L	Meal	Multi-unit
Malt loaf^w^	64 g	920	1.8	M	Snack	Single unit
Pack of crisps (“grab” size)^x^	50 g	1108	1.3	H	Snack	Single unit
Bowl of peanuts^y^	50 g	1338	1.0	H	Snack	Single unit
Macaroni and cheese^z^	410 g	1630	1.9	L	Meal	Single unit
Chicken in black bean sauce with rice^aa^	500 g	2090	1.3	L	Meal	Multi-unit
Cottage pie with broccoli and carrots^ab^	460 g	1175	1.0	VL	Meal	Multi-unit
Ice cream^ac^	400 g	4046	5.3	M	Snack	Single unit
Sandwich roll, snack bar and canned drink “meal deal”^ad^	595 g	4180	1.3	M	Meal	Multi-unit
Pizza with dip^ae^	188 g	3436	1.6	H	Meal	Multi-unit
Quiche with coleslaw^af^	200 g	1547	1.5	M	Meal	Multi-unit
Panini, crisps and fruit juice “meal deal”^ag^	378 g	2796	1.0	M	Meal	Multi-unit

a “Sainsburys”, smooth orange juice carton, as sold.

b “Sainsburys”, two crusty bake snack pork pies, as sold.

c “Sainsburys”, medium cheddar cheese, sliced from packet; “Ritz”, crackers, served from packet.

d “Coca-Cola”, 500 ml container as sold.

e “Sainsburys”, whole milk 1-pint container as sold.

f “Starbucks Coffee Company”, signature hot chocolate, made with full-fat milk, no cream.

g “Sainsburys”, basics crunchy peanut butter, served from jar; “Sainsburys”, sliced white bread, served toasted.

h “Sainsburys”, basics vegetable soup, served from can.

i “Nature’s Finest”, pear, peach and pineapple in juice, served from pack, drained.

j “Muller Light” presented in original container.

k Whole fresh banana, presented with skin (portion size calculated based on flesh only).

l “Sainsburys”, all-butter croissant, served from packet.

m “Sainsburys”, sliced cooked chicken breast; young leaf salad, and Caesar dressing, served from pack/bottle.

n “Weetabix”, breakfast cereal, served from pack (dry); “Sainsburys”, semi-skimmed milk, served from carton.

o “Batchelors”, chicken flavour super noodles, prepared as per manufacturer’s instructions in original container.

p “The Fabulous Bakin’ Boys”, golden oaty flapjack finger, served as sold.

q “Galaxy” chocolate bar, as sold.

r Bakery item, as sold.

s “Jordans”, luxury absolute nut cereal bar, as sold.

t “Longley Farm”, natural cottage cheese, served from container; “Ryvita”, wholegrain rye crispbreads, served from pack.

u “Ginsters”, large sausage roll, presented as sold, in original container.

v “Sainsburys”, baked beans in tomato sauce, served from can; medium cheddar cheese, grated from packet; and sliced white bread, served toasted.

w “Soreen Snack”, malt loaf served with butter, as sold.

x “Walkers”, grab bag ready salted crisps, as sold.

y “Sainsburys”, salted peanuts, served from packet.

z “Sainsburys”, macaroni cheese, served from can.

aa “Morrisons”, chicken in black bean sauce ready meal, served as per instructions.

ab “Sainsburys”, basics cottage pie ready meal, served as per instructions.

ac “Sainsburys”, Madagascan vanilla Devon farmhouse ice-cream tub, presented as sold.

ad “Rustlers”, chicken, bacon and cheese club roll, heated and served as per instructions; “Twix Xtra” chocolate bar and “Coca-Cola”, 330 ml can in original containers.

ae “Domino’s Pizza”, pizza slices, served from box; “Domino’s Pizza”, garlic and herb dip, as sold.

af “Sainsburys”, quiche Lorraine, slice served from box; “Sainsburys” coleslaw, served from pot.

ag “UGO’S”, Bacon, cheese and mustard mayonnaise Panini, prepared as per instructions; “Walkers”, ready salted crisps and “Ocean Spray”, cranberry juice carton, as sold.

**Table 2:** Mean portion estimation ratio (PER) estimated by separate multivariate negative binomial regression models (gender, energy density, unit size category and meal type label, Models 1–4); and jointly, including interaction terms (Model 5). PER = 1 represents correct portion size estimation, PER < 1 under-estimation and PER > 1 over-estimation in relation to FSA reference amounts. A PER of 1.1 for the energy density variable corresponds to a 10% increase in PER per kcal/g (2.4% increase per kJ/g). Clustered data for 32 participants were used, for a total *n* of 1056.

Model	Estimated PER	*P*-value
1. Gender		
Females	0.87	
Males	0.74	0.011

2. Unit number category		
Single unit foods	0.79	
Multi-unit foods	0.83	0.018

3. Meal type label		
Meal or beverage	0.73	
Snacks	0.9	<0.001

4. Energy density (kcal/g)	1.1	<0.001

5. Joint model		
Includes the above variables plus estimated energy, estimated fat, and their interactions:		
Label ^*^ gender	See Fig. 1a	0.009
Estimated energy ^*^ gender	See Fig. 1b	0.09
Energy density ^*^ unit number ∗	See Fig. 1c	0.06
Energy density ^*^ label	See Fig. 1d	<0.001
